# Impacts of negatively charged colloidal clay particles on photoisomerization of both anionic and cationic azobenzene molecules†

**DOI:** 10.1039/d2ra01020h

**Published:** 2022-04-07

**Authors:** Emiko Mouri, Kei Kajiwara, Shuhei Kawasaki, Yusuke Shimizu, Hikaru Bando, Hideki Sakai, Teruyuki Nakato

**Affiliations:** Department of Applied Chemistry, Kyushu Institute of Technology 1-1 Sensui-cho, Tobata Kitakyushu Fukuoka 804-8550 Japan nakato@che.kyutech.ac.jp; Strategic Research Unit for Innovative Multiscale Materials, Kyushu Institute of Technology 1-1 Sensui-cho, Tobata Kitakyushu Fukuoka 804-8550 Japan; Department of Pure and Applied Chemistry, Faculty of Science and Technology, Tokyo University of Science 2641 Yamazaki Noda Chiba 278-8510 Japan

## Abstract

Although smectite-type clays are used as heterogeneous media for photofunctional guest molecules, the guest species are limited to cationic or polar molecules because of the intrinsic negative electric charges of clay particles. Nevertheless, in this study, aqueous clay colloids are reported to affect the photoisomerization kinetics of anionic and cationic azobenzene molecules dissolved in the colloids. Under UV-light irradiation, the clay colloids decelerate *trans*-to-*cis* isomerization, while under visible-light irradiation, the clay colloids accelerate *cis*-to-*trans* isomerization. In addition, the sol–gel transition of clay colloids affects the kinetics. The results considerably expand the applicability of clay colloids as matrixes for functional organic species.

## Abstract 2

Aqueous clay colloids influence the photoisomerization kinetics of both of the anionic and cationic azobenzene molecules although the guest species has been limited to cationic or polar molecules because of the intrinsic negative electric charges of the clay particles.

## Graphical abstract

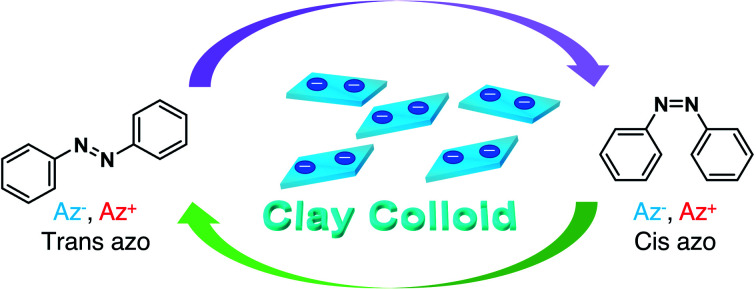

## 1. Introduction

For decades, layered clay minerals have been used as matrixes for organic functional molecules.^1–3^ The photochemically inert, transparent, and colorless aluminosilicate layers of clay minerals serve as ideal media for the immobilization of molecules to exhibit unusual photofunctions.^4^ In particular, smectite-type clay minerals comprising negatively charged layers and charge-compensating interlayer cations immobilize a broad range of guest molecules *via* adsorption onto their silicate layer surfaces or intercalation into their interlayer spaces.^5^ The adsorption and intercalation of the guest molecules occur *via* the exchange of the interlayer cations, usually Na^+^, or dipole interactions between the cations and guest molecules, unless the interlayer spaces or surfaces of the clays are not specially modified.

Thus far, photofunctional molecules investigated for hybridization with smectite-type clays have been limited to cationic or polar molecules.^4^ Strong electrostatic interactions between neighboring clay layers and the cationic guest molecules have been clearly identified to be comprising the origin of the unusual photofunctions.^6^ Anionic photofunctional molecules have been excluded as candidates of the functional molecules hybridized with clays. Although some exceptional cases are reported, such as the use of specific interactions that demonstrated covalent bonding and hydrophobic interactions, complicated experimental processes are required. If the photofunctions of anionic molecules with clay minerals can be easily controlled, then the domain of clay–organic interactions can be considerably expanded.

In this study, the use of aqueous clay colloids as heterogeneous media for anionic photofunctional molecules has been emphasized. Clay colloids are obtained by the delamination of smectite-type clays in an aqueous environment; hydration of the interlayer Na^+^ leads to the infinite swelling of the interlayer spaces, which delaminates the clay layers and forms stable aqueous colloids.^7–9^ Conventional reductionistic recognition of the clay colloids focuses on individual clay particles as the media for functional molecules. In fact, the particles bear anionic charges and attract cationic functional molecules, which exhibit unusual photofunctions.

However, the holistic recognition of clay colloids as continuum reaction media reveals another aspect. A typical representation of this aspect is the sol–gel transition of clay colloids.^7,10–13^ Clay gels are physical hydrogels that are obtained when clay colloids with high clay concentrations are allowed to stand, with the colloids being sols initially. The sol–gel transition is not explained at the molecular level. The gelation of clay colloids does not alter the adsorption behavior of individual clay particles, but it changes the macroscopic property of the colloids. If such a holistic characteristic of clay colloids can contribute to the reactions of photofunctional molecules, then the clay colloids can be used as reaction media to control photofunctions of anionic molecules that are not onto the clay adsorbed particles, in addition to the cationic species.^14^

This idea has prompted our group to investigate the photoisomerization of anionic and cationic azobenzene in aqueous colloids of synthetic hectorite clay (LAPONITE® RD). LAPONITE® RD is a commercially available smectite-type clay mineral with a fine granular appearance and a high surface area of 370 m^2^ g^−1^.^15,16^ It easily forms transparent aqueous colloids, in which nanoparticles with a lateral size of 30 nm and a thickness of 1 nm are dispersed in water.^17^ The photochemistry of azobenzene has been well researched,^18,19^ and its photoisomerization has been investigated in various heterogeneous media.^20–22^ Although several studies on the photochemistry of azobenzene in clay matrixes have been reported,^23–29^ most of them have used solid powders or films in which azobenzene molecules are intercalated to clay minerals. As an exception, the highly retarded photoisomerization of a cationic azobenzene on colloidal clay particles has been reported and ascribed to electrostatic immobilization by negatively charged clay surfaces.^30^ In this work, clay colloids are reported to alter the reaction rates of anionic and cationic azobenzene. The sol–gel transition of clay colloids also affects the reaction rates. Although clay colloids exert different effects on anionic and cationic azobenzene, this study is the first example demonstrating the controllability of the photofunctions of anionic molecules by aqueous clay colloids.

**Fig. 1:**
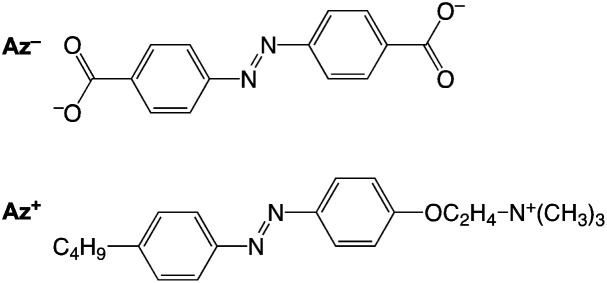
Molecular structure of Az^−^ and Az^+^.

Azobenzene-4,4′-dicarboxylate (Az^−^) and 4-butylazobenzene-4′-(oxyethyl)trimethylammonium (Az^+^)^31^ were used as the anionic and cationic azobenzene species, respectively (Fig. 1). LAPONITE® RD (Rockwood Additives Ltd) was used as the clay source. A clay colloid sample was prepared by the gradual addition of clay powders to water, followed by mixing with an aqueous solution of an azobenzene species. Some colloid samples underwent sol–gel transition after the samples were allowed to stand under ambient conditions in the dark. Photoisomerization of the azobenzene molecules in the colloids of sols and gels was carried out under UV- and visible-light irradiations for the *trans*-to-*cis* and *cis*-to-*trans* isomerization, respectively.

## 2. Experiments

### 2.1 Materials

Azobenzene-4,4′-dicarboxylic acid (Az^−^) (Tokyo Chemical Industry Co., Ltd, Tokyo, Japan) and LAPONITE® RD (Rockwood Additives Ltd) were used as obtained. 4-Butylazobenzene-4′-(oxyethyl)trimethylammonium (Az^+^) was synthesized according to a method reported previously.^31^

### 2.2 Sample preparation

A typical clay dispersion sample was prepared as follows. First, clay (0.33–0.77 g) was slowly dispersed into 50 mL of stirred Mill-Q water little by little. Next, the clay dispersion was stirred for 30 min. This clay dispersion (20 mL) was mixed with 1.1 × 10^−4^ mol L^−1^ Az solution (2 mL), which was prepared as described below. The mixed solution was stirred for 30 min to obtain a clay colloid with a concentration of 1.0 × 10^−5^ mol L^−1^ Az. For the Az^−^ solution, 50 mL of 2 mol L^−1^ NaOH (0.1 mmol) was mixed with 0.0074 g Az^−^ (0.0274 mmol) to ensure the dissociation of the carboxylic acid. Subsequently, it was diluted to give a concentration of 1.1 × 10^−4^ mol L^−1^. For the Az^+^ solution, additional treatment was not conducted to prepare the 1.1 × 10^−4^ mol L^−1^ aqueous solution because the Az^+^ compound of the alkyl ammonium salt dissociated perfectly in water.

### 2.3 Photoisomerization

The sample solution in a quartz cell (10 mm thickness) was subjected to UV- and vis-light irradiation. At a 10 min interval until the spectrum was unchanged by additional irradiation, the UV-vis spectra of the sample were recorded on a V-570 spectrometer (Jasco, Tokyo, Japan). For *trans*-to-*cis* photoisomerization experiments, a high-pressure UV lamp (SX-UI251HQ, 250 W, Ushio, Tokyo, Japan) with a long-wavelength (>400 nm) cut filter and a water filter was used. For *cis*-to-*trans* photoisomerization experiments, we used a Xe lamp (SX-UI501XQ, 500 W Ushio, Tokyo, Japan) with an L-42 cut filter (<400 nm), ND40, ND50, and a water filter.

### 2.4 Data analysis

UV-vis spectra of the samples containing clay revealed scattering in the long-wavelength range. The background assuming Rayleigh scattering was subtracted from the spectra. More specifically, in this case, the spectra were fitted with Abs = *α* + *βλ*^−4^ in the wavelength range of *λ* > 600 nm and additional absorbance at the isosbestic point, and the values obtained by Abs = *α* + *βλ*^−4^ were subtracted from the spectra, where *λ* is the wavelength, and *α* and *β* are fitted constants.

To estimate the photoisomerization rate, the peak top values are tabulated along with the irradiation time. For the data of UV irradiation (*trans*-to-*cis*), for each sample, absorbance values are normalized by the absorbance before irradiation. The normalized absorbance is denoted as *A*_*t*_, where *t* is the irradiation time. Hence, *A*_0_ is always 1 by definition. Afterward, ln(*A*_0_ − *A*_inf_/*A*_*t*_ − *A*_inf_) is calculated, where *A*_inf_ is the asymptotic value of *A* at an infinite irradiation time, *i.e.*, 0.6 for Az^−^ and 0.2 for Az^+^ systems.

From the slope of the plot of ln(*A*_0_ − *A*_inf_/*A*_*t*_ − *A*_inf_) against *t*, the rate constant *k* can be estimated. The rate constant can be estimated on the basis of the data from 0 min to 20 min because the plot is confirmed to be linear in this range.

For the data of visible irradiation (*cis*-to-*trans*), absorbance values are normalized by the absorbance at infinite irradiation for each sample, *i.e.*, *A*_inf_ is defined as 1. Subsequently, ln(*A*_0_ − *A*_inf_/*A*_*t*_ − *A*_inf_) is plotted against *t*, and the rate constant *k* is obtained from the slope of the plots. The rate constant is estimated on the basis of the data from 0 min to 20 min because the plot is confirmed to be linear in this range.

## 3. Results and discussion

**Fig. 2:**
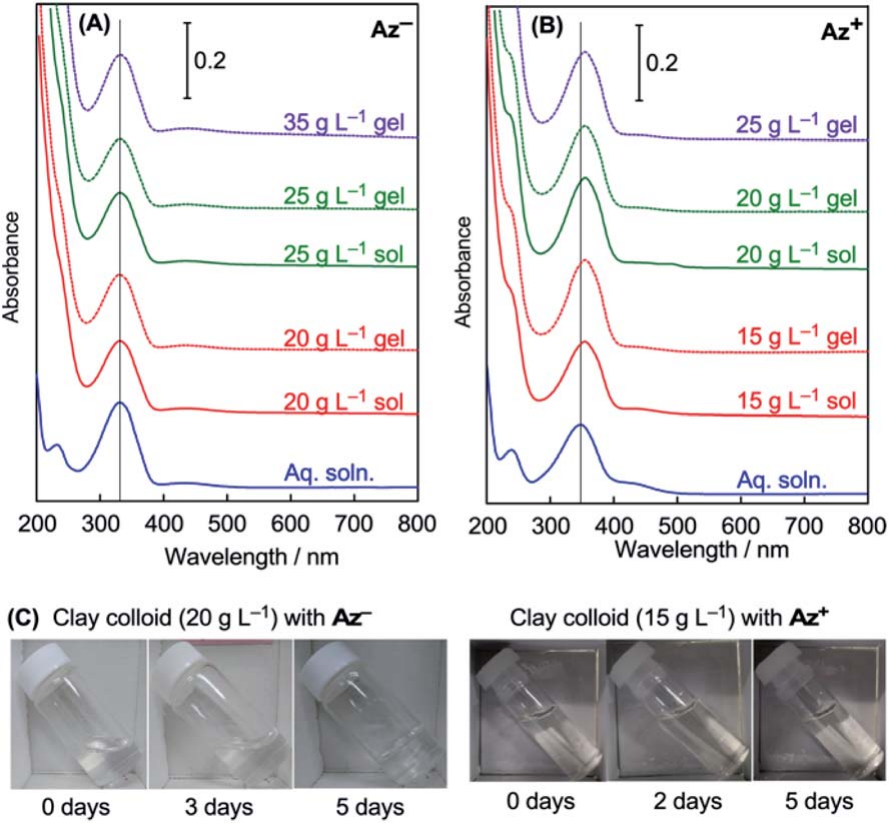
UV-vis absorption spectra of (A) Az^−^ and (B) Az^+^ in the aqueous solution and clay colloids, and (C) photographs of the sol–gel transition of the clay colloids containing Az^−^ or Az^+^. The vertical lines in panels (A) and (B) indicate *λ*_max_ of the aqueous solutions of Az^−^or Az^+^.

Aqueous colloids of smectite-type clay minerals undergo sol–gel transition at sufficiently high clay concentrations, at which fluid clay colloids (clay sols) change to physical hydrogels (clay gels). A clay colloid with a higher clay concentration tends to be gelated within a short period to form a stiffer gel, although the gelation behavior is affected by the coexisting solutes in the colloid. Table 1 summarizes gelation periods of the clay colloids with different clay concentrations at a constant azobenzene concentration examined in this study. For the colloids with Az^−^, gelation occurs at a clay concentration of greater than 20 g L^−1^, and the sample with a clay concentration of 35 g L^−1^ rapidly undergoes gelation during sample preparation. For the colloids with Az^+^, gelation occurs at a clay concentration of greater than 15 g L^−1^, and the sample with a clay concentration of 25 g L^−1^ is obtained only as a gel. Fig. 2 shows photographs of the typical colloid samples exhibiting sol–gel transition.

**Table 1:** Sample status (sol or gel), gelation period, and *λ*_max_ of the azobenzene species in the samples for the clay colloid samples

Azobenzene species	Clay conc./g L^−1^	Sample status	Period for the gelation	*λ*_max_ of the absorption/nm
Az^−^	0	Aq. soln	—	331
Az^−^	20	Clay sol	—	331
Az^−^	20	Clay gel	5 d	331
Az^−^	25	Clay sol	—	331
Az^−^	25	Clay gel	1–2 d	331
Az^−^	35	Clay gel	10 min	331
Az^+^	0	Aq. soln	—	348
Az^+^	15	Clay sol	—	355
Az^+^	15	Clay gel	4–5 d	355
Az^+^	20	Clay sol	—	355
Az^+^	20	Clay gel	2–5 d	355
Az^+^	25	Clay gel	1–2 d	355

**Fig. 3:**
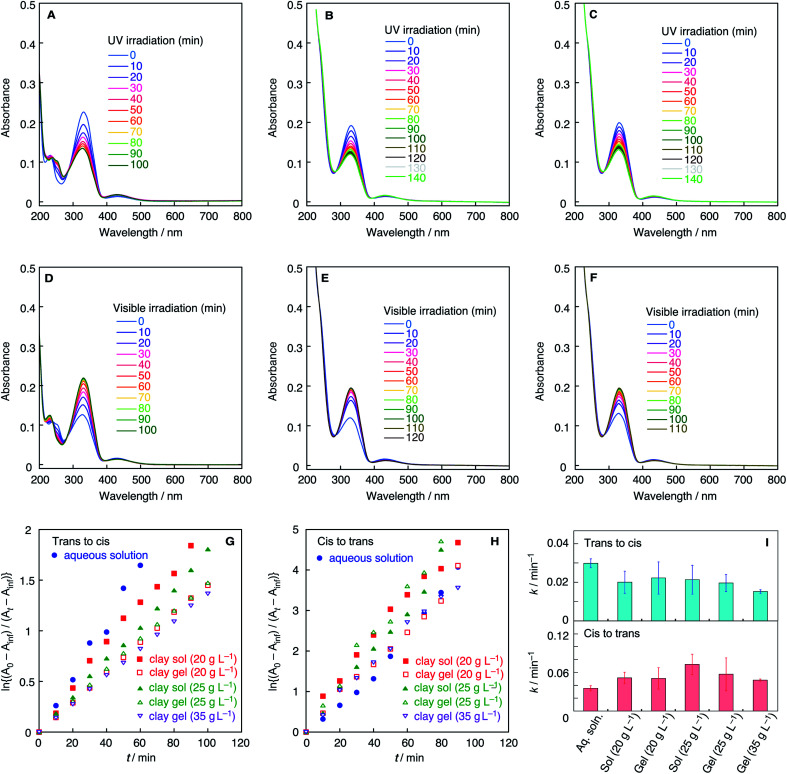
UV-vis spectra of Az^−^ during *trans*-to-*cis* isomerization in (A) aqueous solution, (B) clay sol (20 g L^−1^), and (C) clay gel (20 g L^−1^) upon irradiation of UV light. UV-vis spectra of Az^−^ during *cis*-to-*trans* isomerization in (D) aqueous solution, (E) clay sol (20 g L^−1^), and (F) clay gel (20 g L^−1^) upon irradiation of visible light. (G) First-order kinetics plots of *trans*-to-*cis* isomerization of Az^−^ in the aqueous solution and clay sols and gels. (H) First-order kinetics plots of *cis*-to-*trans* isomerization of Az^−^ in the aqueous solution and clay sols and gels. (I) First-order rate constants of *trans*-to-*cis* and *cis*-to-*trans* isomerization of Az^−^ in the aqueous solution and clay sols and gels.

**Fig. 4:**
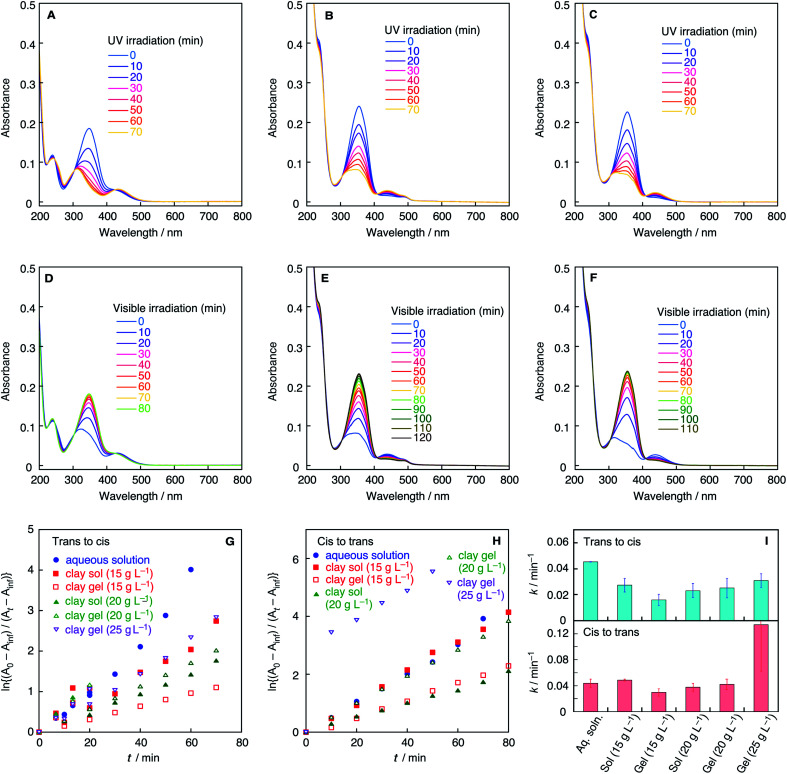
UV-vis spectra of Az^+^ during *trans*-to-*cis* isomerization in (A) aqueous solution, (B) clay sol (20 g L^−1^), and (C) clay gel (20 g L^−1^) upon irradiation of UV light. UV-vis spectra of Az+ during *cis*-to-*trans* isomerization in (D) aqueous solution, (E) clay sol (20 g L^−1^), and (F) clay gel (20 g L^−1^) upon irradiation of visible light. (G) First-order kinetics plots of *trans*-to-*cis* isomerization of Az^−^ in the aqueous solution and clay sols and gels. (H) First-order kinetics plots of *cis*-to-*trans* isomerization of Az^+^ in the aqueous solution and clay sols and gels. (I) First-order rate constants of *trans*-to-*cis* and *cis*-to-*trans* isomerization of Az^+^ in the aqueous solution and clay sols and gels.

The microenvironment of the azobenzene molecules in clay colloids is evaluated by visible spectroscopy. Generally, the adsorption of cationic dyes onto negatively charged smectite-type clay particles has been known to be detected by absorption spectra of dyes. Fig. 2 shows the visible absorption spectra of clay colloids involving the Az^−^ or Az^+^ dyes before photoirradiation. The azobenzene molecules are present as the *trans* isomer, and Table 1 lists their wavelengths at the absorption maxima (*λ*_max_). Az^−^ in the clay colloids exhibits the same *λ*_max_ (331 nm) as that of an aqueous solution, whereas Az^+^ exhibits *λ*_max_ (355 nm) red-shifted from that of an aqueous solution (348 nm). The results indicate that Az^+^, and not Az^−^, is adsorbed on the clay particles. However, because clay sols and gels exhibit the same *λ*_max_ for Az^−^ and Az^+^, the sol–gel transition of clay colloids does not affect the spectroscopically probed microenvironment of both azobenzene dyes. Photoisomerization reactions of Az^−^ and Az^+^ in the clay colloids were conducted under UV and visible-light irradiations for the *trans*-to-*cis* and *cis*-to-*trans* isomerization, respectively. Fig. 3 and 4 show variations of visible absorption spectra of typical samples. UV irradiation causes *trans*-to-*cis* isomerization in Az^−^ and Az^+^ systems, as indicated by the decrease in the characteristic π–π* absorption (331 nm for Az^−^ and 348 or 355 nm for Az^+^; see Table 1) of the *trans* isomer. Photostationary states are obtained for most of the samples within 140 min; *trans*Az^−^ and Az^+^ decrease by 40% and 80%, respectively, irrespective of the coexistence of clay particles. *Cis*-to-*trans* isomerization is observed under visible irradiation, and >95% of the initial amount of the *trans* isomer is recovered.

The time course of photoisomerization is analyzed by plotting ln{(*A*_0_ − *A*_inf_)/(*A*_*t*_ − *A*_inf_)} against *t*, where *A*_0_, *A*_inf_, and *A*_*t*_ are the normalized absorbance of the *trans* isomer before the reaction, normalized absorbance after the reaction for a sufficiently long time, and normalized absorbance for time *t*, respectively, for *trans*-to-*cis* isomerization.^32,33^ In *cis*-to-*trans* isomerization, the procedure is basically the same but the definition of *A*_0_ and *A*_inf_ are different as stated previously. Fig. 3G, H, 4G and H show the plots of typical samples of Az^−^ and Az^+^ systems. The time courses except for the *cis*-to-*trans* isomerization of the Az^+^ system are basically fitted by straight lines, indicative of first-order kinetics. Comparison of aqueous colloids and clay colloids indicates that the coexistence of clay particles does not alter the reaction order of each photoisomerization reaction with each azobenzene species. Fig. 3I and 4I summarize the rate constants estimated from plots of the variation of absorbance values; they are estimated from the initial 20 min where the plots are not considerably deviated even though the reactions do not obey first-order kinetics. In addition, the fluctuation of the reaction time courses and hence the rate constants is mentioned. This is remarkable for the clay colloids in the sol and gel states, especially for Az^−^ in the colloids. Thus, rate constants are estimated as averages of several runs after the *Q*-test. Table S1† summarizes rate constants with standard deviations, and Fig. S1–S4 show all of the time courses adopted for the estimations.

These results demonstrate the effects of colloidal clay particles on the photoisomerization kinetics of Az^−^ and Az^+^ dyes. For anionic and cationic azo dyes, the clay colloids decelerate *trans*-to-*cis* isomerization, whereas they mostly accelerate *cis*-to-*trans* isomerization; some exceptions are observed in the Az^+^ system. Although the clay particles are negatively charged, the clay colloids behave as heterogeneous reaction media that typically affect the photochemical reaction in aqueous media in a similar manner irrespective of the electric charge of the reactant molecules. Retardation of the reactions of cationic azobenzene reported in a previous study^30^ is not observed. This is rationalized by the amphiphilic nature of Az^+^ used herein because the hydrophobic tail of the Az^+^ molecule is not confined near the clay surfaces to weaken the electrostatic interactions, which is different from the strong interactions realized in the previously reported study.

On the other hand, detailed conditions of the colloids exemplified by the clay concentration and sol–gel transition render different effects on Az^−^ and Az^+^. A clear difference is observed for the gel samples with a high clay concentration. For Az^−^, either for *trans*-to-*cis* or *cis*-to-*trans* reaction, isomerization is most decelerated for clay gels with a high clay concentration among clay-containing systems. In contrast, for Az^+^, *trans*-to-*cis* and *cis*-to-*trans* isomerization reactions are most accelerated for clay gels with a high clay concentration among clay-containing systems.

In this study, however, it was difficult to reduce further the fluctuation of the isomerization kinetics to ensure perfect reproducibility of the clay concentration dependence. This would be due to the nonergodic property of the clay colloids as exemplified by the slow sol–gel transition. This characteristic of clay colloids can result in large fluctuations of the rate constants observed in Fig. 3I and 4I. However, for Az^−^ in clay sols, *trans*-to-*cis* and *cis*-to-*trans* isomerization reactions are accelerated with the increase in the clay concentration. In addition, the *trans*-to-*cis* reaction in the clay gels is decelerated with the increase in the clay concentration, and the *cis*-to-*trans* isomerization rate is maximized at an intermediate clay concentration. Alternatively, we do not exclude the interpretation that the photoisomerization reaction rates, especially *trans*-to-*cis*, of clay sols and gels with a clay concentration of less than 25 g L^−1^ are almost the same.

In contrast, the reactions of Az^+^ exhibit almost opposite dependencies on the former description of the Az^−^ system. The *trans*-to-*cis* and *cis*-to-*trans* isomerization reactions are decelerated with the increase in the clay concentration of clay sols, whereas they are accelerated at higher clay concentrations of clay gels. Effects of such detailed sample conditions on the photoisomerization rates for Az^−^ and Az^+^ would suggest the contribution of different detailed mechanisms of the reaction control for Az^−^ and Az^+^. At the moment, the obtained results do not clearly explain the mechanisms, but we assume that microscopic and macroscopic aspects to the mechanism are possible. The microscopic aspect is that electrostatic attractive interactions between anionic dye species and cationic charges at the edge of the clay particles exist.^34^ The macroscopic aspect is that rheological properties change according to the clay component. Moreover, the lack of clear trends in the sol–gel transition of clay colloids suggests that clay sols and gels can be recognized as continuum media that are independent of each other although a concrete model is not achieved yet. A difference in the polarities of the reaction media reported for the kinetics of azobenzene photoisomerization in homogeneous solutions^35,36^ does not explain the obtained results because the clay sols and gels exhibit the same solvent polarity, albeit with different photoisomerization kinetics.

## 4. Conclusions

In conclusion, the obtained results demonstrate that aqueous colloids of smectite-type clays, in which colloidal clay particles bear negative electric charges, affects the photoisomerization of anionic and cationic azobenzene. The results reveal unexpected effects of clay particles as matrixes or a heterogeneous medium of photofunctional molecules. This is a novel insight into clay–organic interactions, which is explained by molecular-level attractive electrostatic interactions between the organic molecules and clay particles.

## Author contributions

Emiko Mouri: supervision, data analysis, writing, and editing. Kei Kajiwara: sample preparation and data collection for the Az^−^ system. Shuhei Kawasaki: sample preparation and data collection for the Az^+^ system. Yusuke Shimizu: sample preparation and data collection. Hikaru Bando: sample preparation and data collection. Hideki Sakai: supervision for synthesizing Az^+^ and review. Teruyuki Nakato: conceptualization, supervision, original draft writing, and editing.

## Conflicts of interest

There are no conflicts to declare.

## Supplementary Material

RA-012-D2RA01020H-s001
