# Supplementary material for: Impacts of negatively charged colloidal clay particles on photoisomerization of both anionic and cationic azobenzene molecules
Source: RSC Adv. 2022 Apr 7;12(17):10855–61. doi: 10.1039/d2ra01020h (PMC8988266; doi:10.1039/d2ra01020h)
Supplement: RA-012-D2RA01020H-s001 [file RA-012-D2RA01020H-s001.pdf]

Electronic Supplementary Material (ESI) for RSC Advances.
This journal is © The Royal Society of Chemistry 2022

Electronic Supplementary Information

Impacts of negatively charged colloidal clay particles on photoisomerization of both of the anionic and cationic azobenzene molecules

Emiko Mouri,[a,b] Kei Kajiwara,[a] Shuhei Kawasaki,[a] Yusuke Shimizu,[a] Hikaru Bando,[a] Hideki Sakai,[c] Teruyuki Nakato,[a,b]*

[a] Department of Applied Chemistry, Kyushu Institute of Technology, 1-1 Sensui-cho, Tobata, Kitakyushu, Fukuoka 804-8550, Japan

[b] Strategic Research Unit for Innovative Multiscale Materials, Kyushu Institute of Technology, 1-1 Sensui-cho, Tobata, Kitakyushu, Fukuoka 804-8550, Japan

[c] Department of Pure and Applied Chemistry, Faculty of Science and Technology, Tokyo University of Science, 2641 Yamazaki, Noda, Chiba 278-8510, Japan

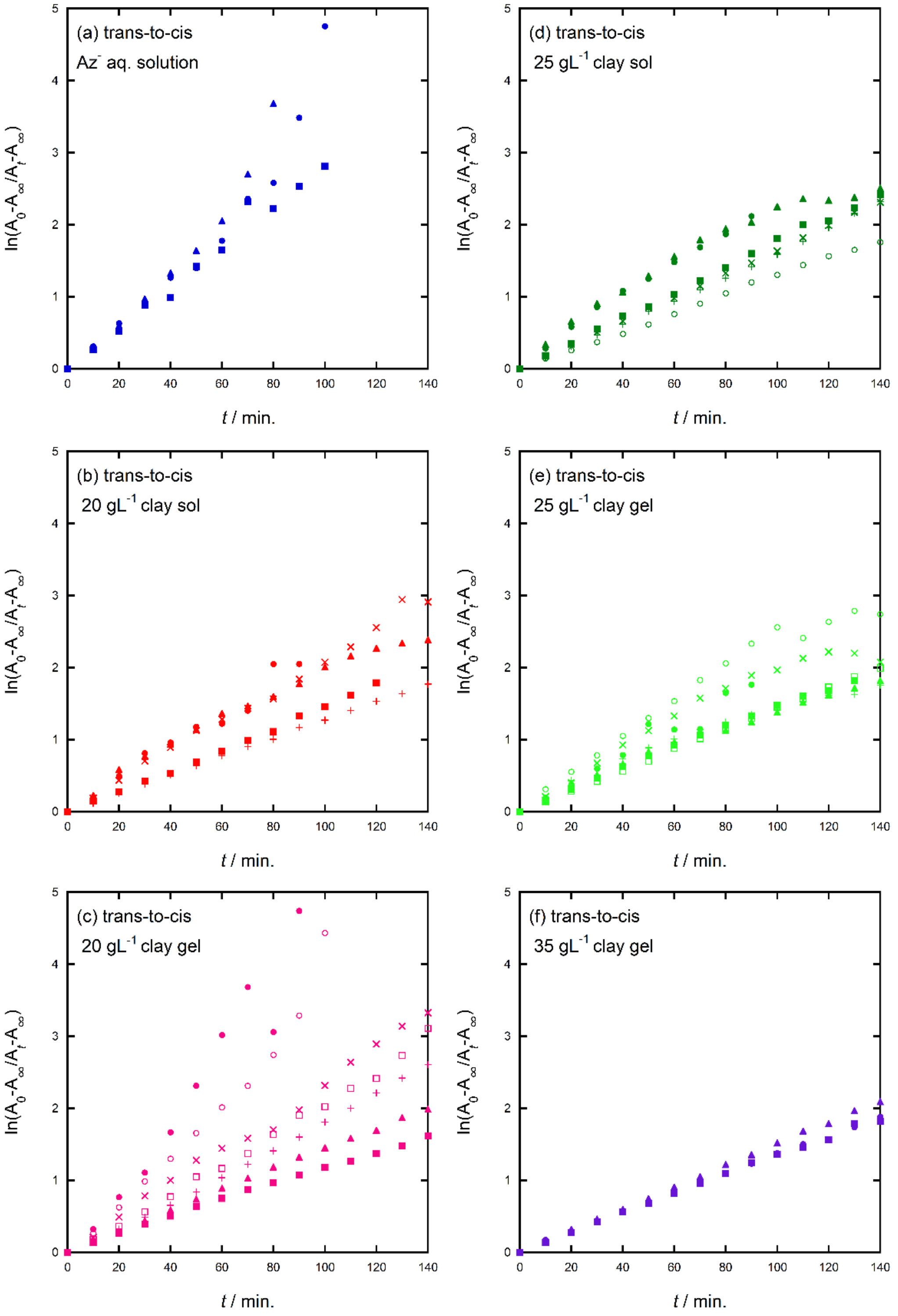

Figure S1. First-order plots against UV light irradiation time for Az^- systems from trans to cis form. (a) Az^- aqueous solution, (b) 20 g L^{-1} clay sol with Az^-, (c) 20 g L^{-1} clay gel with Az^-, (d) 25 g L^{-1} clay sol with Az^-, (e) 25 g L^{-1} clay gel with Az^-, (f) 35 g L^{-1} clay gel with Az^-.

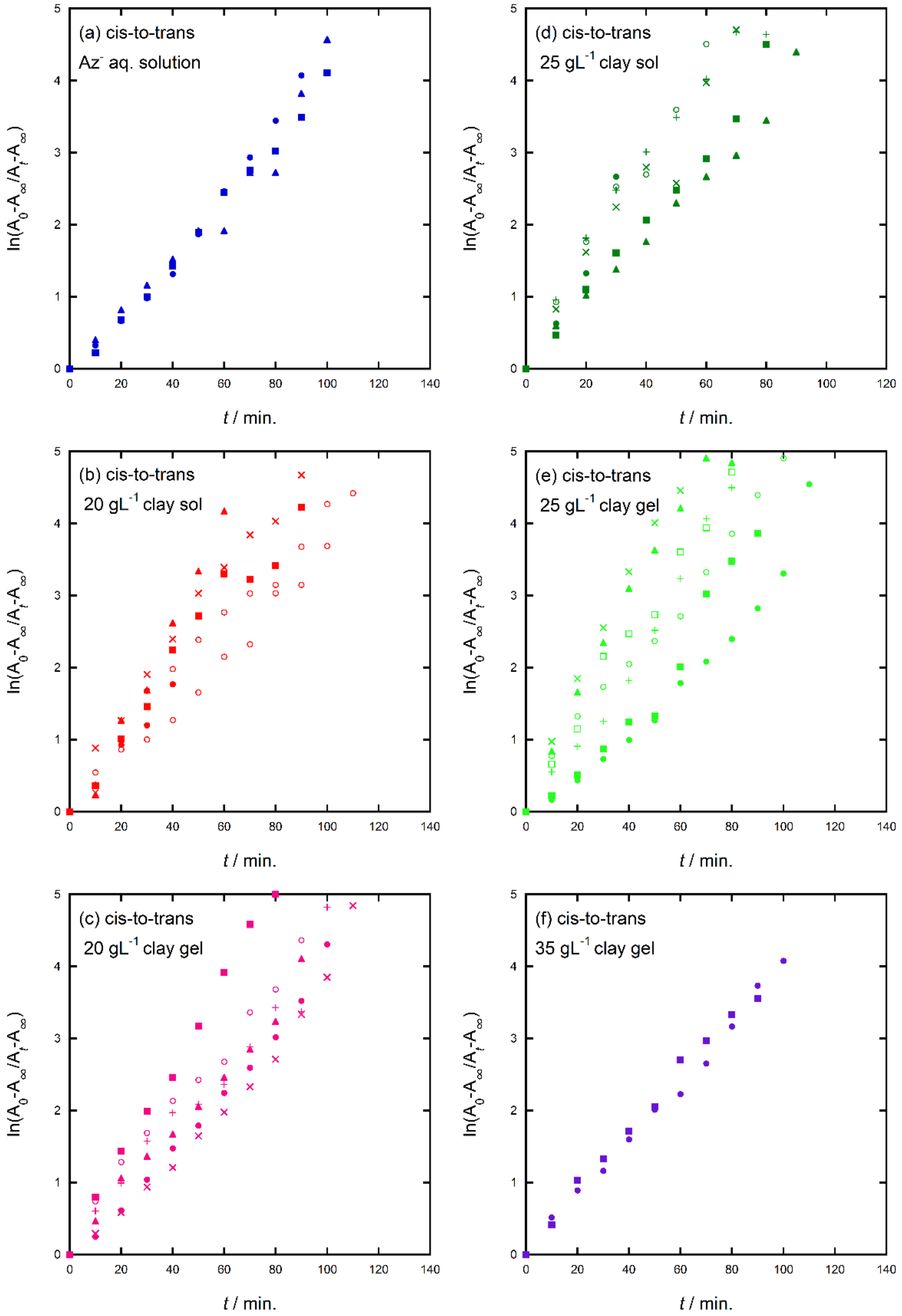

Figure S2. First-order plots against visible light irradiation time for Az^- systems from cis to trans form. (a) Az^- aqueous solution, (b) 20 g L^{-1} clay sol with Az^-, (c) 20 g L^{-1} clay gel with Az^-, (d) 25 g L^{-1} clay sol with Az^-, (e) 25 g L^{-1} clay gel with Az^-, (f) 35 g L^{-1} clay gel with Az^-.

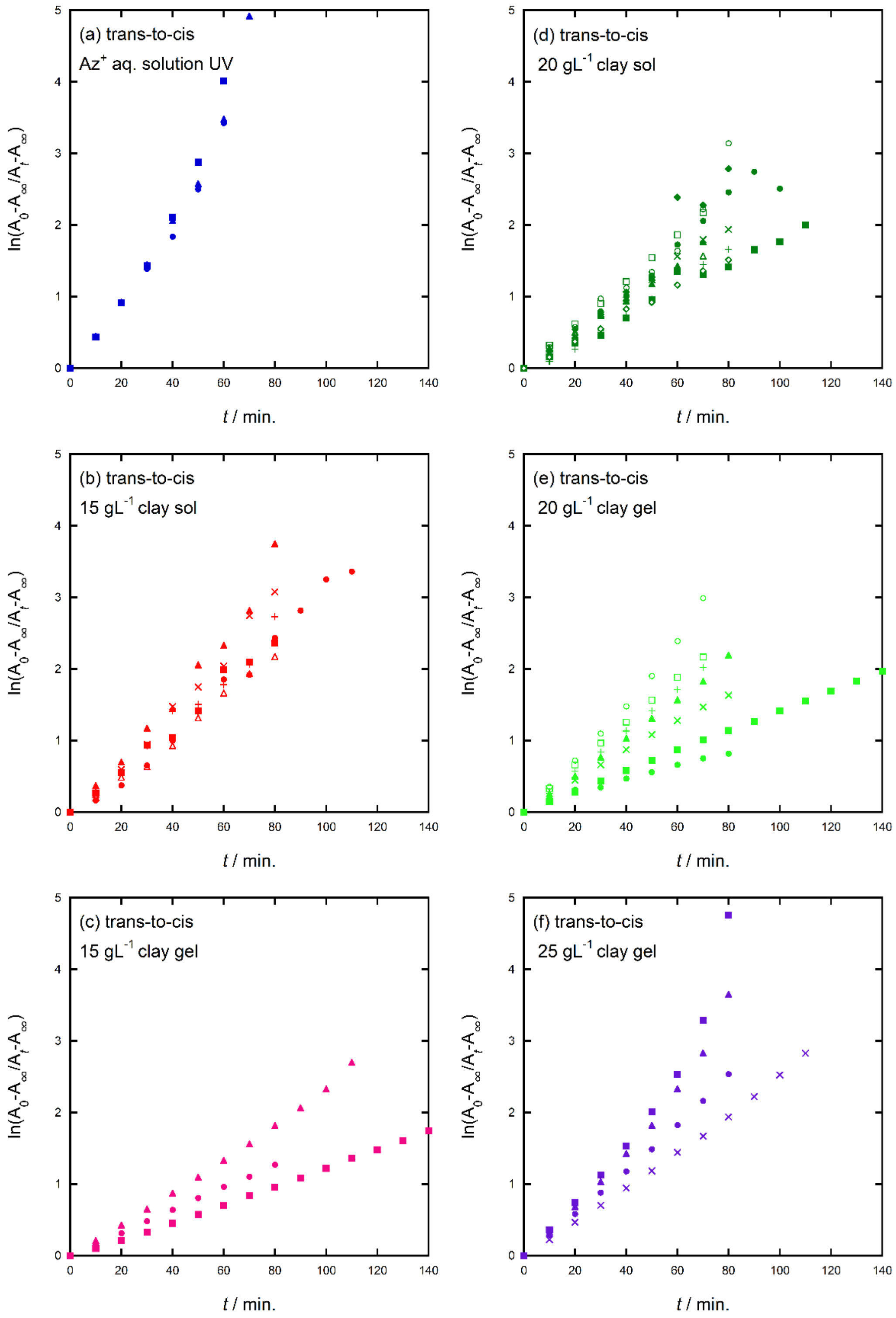

Figure S3. First-order plots against UV light irradiation time for Az^+ systems from trans to cis form. (a) Az^+ aqueous solution, (b) 15 g L^{-1} clay sol with Az^+, (c) 15 g L^{-1} clay gel with Az^+, (d) 20 g L^{-1} clay sol with Az^+, (e) 20 g L^{-1} clay gel with Az^+, (f) 25 g L^{-1} clay gel with Az^+.

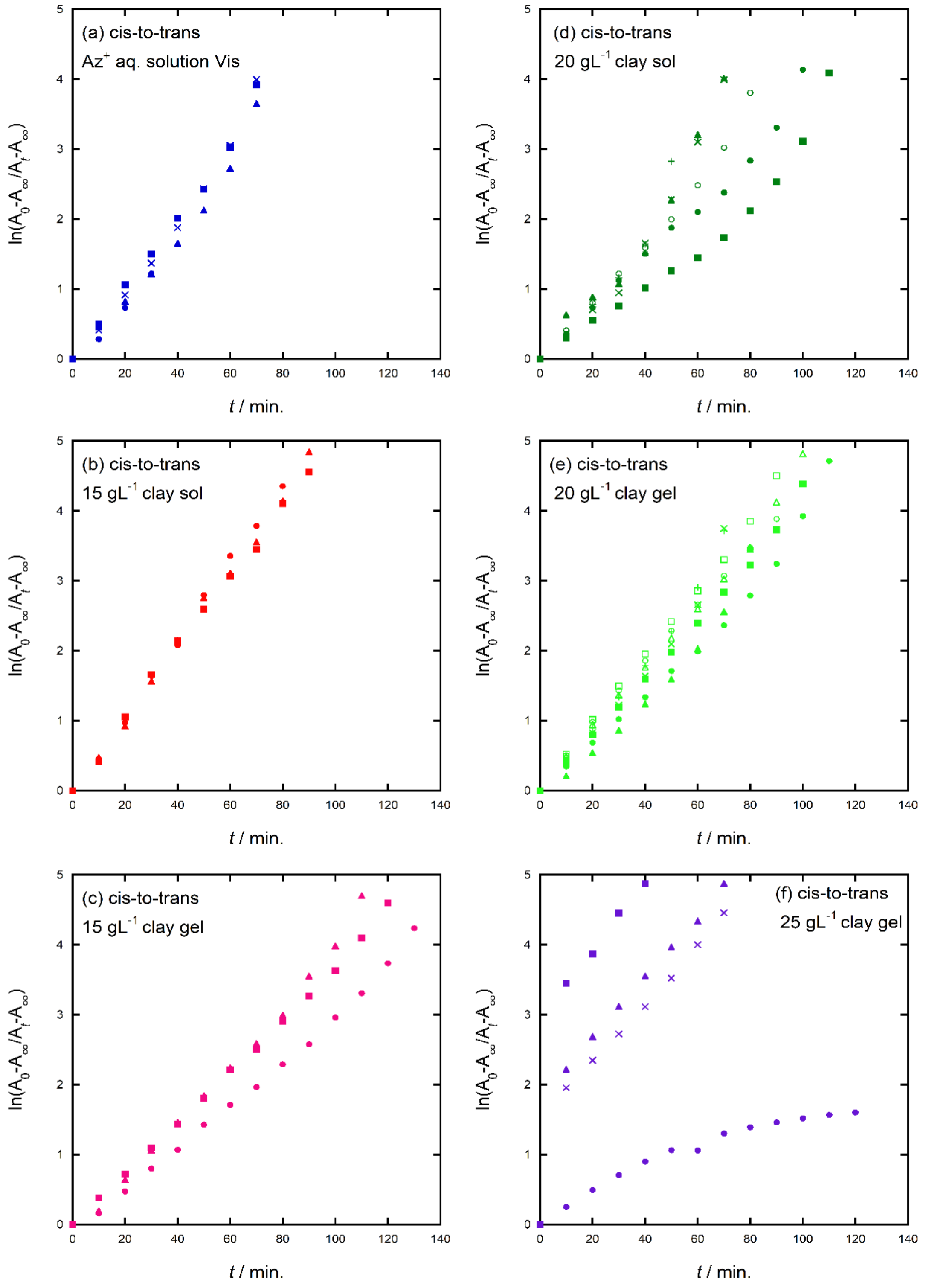

Figure S4. First-order plots against visible light irradiation time for Az^{+} systems from cis to trans form. (a) Az^{+} aqueous solution, (b) 15 g L^{-1} clay sol with Az^{+}, (c) 15 g L^{-1} clay gel with Az^{+}, (d) 20 g L^{-1} clay sol with Az^{+}, (e) 20 g L^{-1} clay gel with Az^{+}, (f) 25 g L^{-1} clay gel with Az^{+}.

Table S1. First-order rate constants for trans-to-cis and cis-to-trans photoisomerization of azobenzene dissolved in the clay colloids.

Azobenzene species	Clay concentration / g L^{-1}	Sample status	Trans-to-cis rate constant / min^{-1}	Cis-to-trans rate constant / min^{-1}
**Az^{-}**	0	Aqueous solution	0.0289 ± 0.0029	0.0351 ± 0.0040
	20	Clay sol	0.0198 ± 0.0058	0.0512 ± 0.0089
	20	Clay gel	0.0219 ± 0.0083	0.0503 ± 0.0167
	25	Clay sol	0.0209 ± 0.0074	0.0722 ± 0.0160
	25	Clay gel	0.0192 ± 0.0043	0.0567 ± 0.0255
	35	Clay gel	0.0149 ± 0.0009	0.0477 ± 0.0017
**Az^{+}**	0	Aqueous solution	0.0454 ± 0.0003	0.0435 ± 0.0063
	15	Clay sol	0.0269 ± 0.0052	0.0483 ± 0.0017
	15	Clay gel	0.0158 ± 0.0044	0.0294 ± 0.0058
	20	Clay sol	0.0230 ± 0.0055	0.0374 ± 0.0061
	20	Clay gel	0.0250 ± 0.0072	0.0418 ± 0.0079
	25	Clay gel	0.0307 ± 0.0052	0.1333 ± 0.0712
